# Templated microwave synthesis of luminescent carbon nanofibers†

**DOI:** 10.1039/c7ra13383a

**Published:** 2018-04-06

**Authors:** Clara Deeney, Suxiao Wang, Samir A. Belhout, Aoife Gowen, Brian J. Rodriguez, Gareth Redmond, Susan J. Quinn

**Affiliations:** School of Chemistry, University College Dublin Dublin 4 Ireland gareth.redmond@ucd.ie susan.quinn@ucd.ie; School of Biosystems and Food Engineering, University College Dublin Dublin 4 Ireland; School of Physics, University College Dublin Dublin 4 Ireland; School of Chemistry and Materials Science, Hubei University Wuhan 430062 China

## Abstract

Carbon based nanomaterials offer the potential to provide solutions to key technological challenges. This work describes the preparation of luminescent carbon nanofibers by template-assisted microwave pyrolysis of environmentally friendly precursors, citric acid and polyethyleneimine, in aqueous solution. SEM reveals a dense forest of vertically aligned cylindrical carbon nanofibers with an average diameter of *ca.* 200 nm, which are shown by TEM to be amorphous. Compositional analysis indicated the incorporation of amino and pyrrolic nitrogen, and carbon–oxygen moieties. These species contribute to UV light absorption with an absorption shoulder and tail towards visible wavelengths. UV excitation gave visible (blue) emission at *ca.* 450 nm with a quantum yield of *ca.* 5%; emission decay under pulsed excitation was predominantly mono-exponential with a lifetime of *ca.* 1 ns. The emission maximum is largely excitation wavelength independent suggesting the involvement of citrazinic acid-type functionalities in the fiber photophysics. Reversible pH-dependent excitation and emission behaviour was observed, with maximum emission at *ca.* pH 7. Nanofiber emission was also quenched in aqueous solutions of metal cations, in a concentration-dependent manner. Single nanofiber emission intensity was quite stable under continuous excitation permitting single fiber quenching-based metal ion detection whereby a significant (>90%) and prompt (sub-10 s) quenching was observed upon exposure to sub-millimolar Fe(iii) solutions. The introduction of these new 1D luminescent carbon nanofibers offers the potential for exciting developments across a range of applications.

## Abstract 2

This work describes the preparation of luminescent carbon nanofibers by template-assisted microwave pyrolysis of environmentally friendly precursors in aqueous solution.

## Graphical abstract

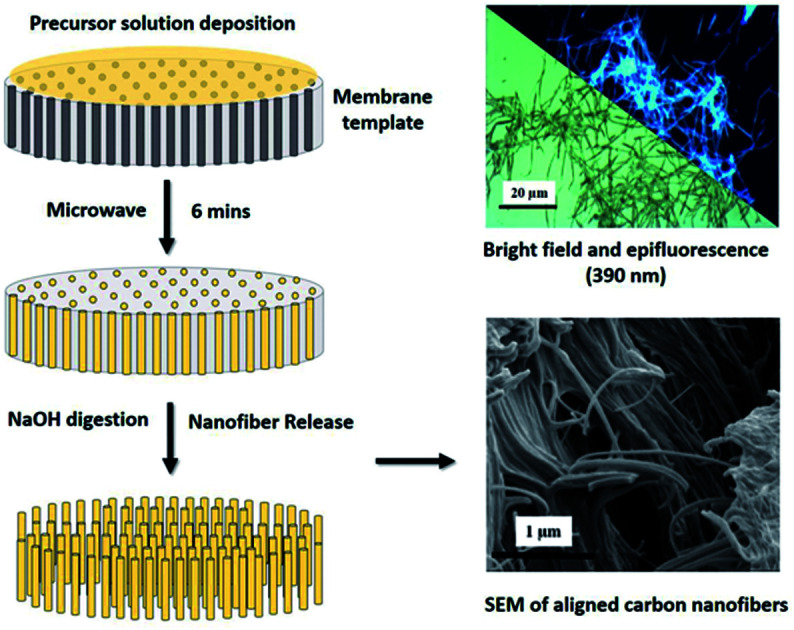

## Introduction

In response to the demand for greener technologies, carbon based functional materials, including carbon nanomaterials^1–4^ are increasingly finding application in areas as diverse as sensing, imaging, catalytic supports and energy storage devices.^5–7^ One-dimensional (1D) carbon nanotubes, display a wide range of optical and electronic properties, which have been exploited for biological and technological applications.^8–12^ Traditionally, carbon and carbon rich polymeric nanofibers and 1D fibers are of considerable interest due to their potential as catalyst supports,^13,14^ supercapacitors,^15^ and energy storage materials.^16,17^ Carbon 1D nanofibers have been prepared by carbonization of oriented polymers and biopolymers.^18,19^ An alternative approach to the formation of carbon micro- and nano-fibers is to use well-defined channels in porous membranes as templates for appropriate precursor materials.^20^ In this manner, porous alumina membranes containing anodically etched pores have been used to prepare pyrolysed carbon nanofibers.^16,21,22^

However, while the preparation of such non-luminescent carbon nanofibers is well established, the preparation of luminescent carbon 1D fibers in the micro- and nano-scale is yet to be reported. For example, luminescent carbon nanomaterials such as semiconducting single walled nanotubes,^23,24^ nanodiamonds^25^ and Cdots^26–28^ are attractive materials for sensing and imaging applications and of these the latter are particularly noteworthy. Luminescent carbon dots (Cdots) are currently stimulating a revolution in carbon based photonics and optoelectronics.^29–33^ Discovered in 2004, as a by-product of nanotube synthesis,^26^ Cdots were soon prepared deliberately *via* laser ablation.^27^ Since then Cdots have been the subject of intense study due to their excellent luminescent properties,^32,34^ biocompatibility^35^ and accessibility through facile synthesis from diverse, inexpensive and environmentally friendly precursors.^31^

The range of synthetic methods used to prepare Cdots include laser ablation,^27^ thermal pyrolysis^36^ by solvothermal^34,37^ and hydrothermal^38,39^ synthesis, as well as electrochemical^40,41^ and microwave assisted pyrolysis.^35,42–44^ Precursors include, alcohols,^40^ bio-organic acids,^36^ amino acids,^44,45^ biomass^46^ and waste materials.^47^ Notably Cdots have also been isolated from candle soot^48^ and extracted from foods.^49^ Access to luminescent Cdots of uniform size has recently been achieved by pyrolysis of a citric acid precursor in a mesoporous silica templates.^50–52^

These methods provide access to Cdots whose tunable luminescence spans the visible spectrum. The nature of Cdot luminescence has been the subject of significant debate.^32,33^ The interplay between surface states and electronic transitions within a crystalline or highly pyrolysed core has emerged as a key factor in the behaviour.^.^ Importantly, the luminescence of many Cdot systems has been found to be very sensitive to surface binding interactions which have led to considerable interest in sensing.^31^

In this work, we leverage the developments in Cdot synthesis and templated 1D nanomaterial processing to describe the first preparation of luminescent carbon nanofibers *via* a template-assisted microwave synthesis. Microwave synthesis has the advantages of rapid heating and upscaling potential, and this has permitted Cdots to be easily made under almost industrial conditions.^42,53^ The synthesis uses environmentally friendly precursors, citric acid and polyethyleneimine (PEI), that have been previously used to prepare Cdots.^36^ Using this approach, dense forests of vertically aligned cylindrical carbon nanofibers, with an average diameter of *ca.* 200 nm, could be efficiently prepared. Compositional analysis of the fibers indicated the presence of aliphatic and graphitic carbon with incorporation of amino and pyrrolic nitrogen as well as carbon-oxygen moieties. The nanofibers absorbed UV light with a tail towards visible wavelengths and the resulting emission varied in intensity with excitation wavelength while the spectral maximum was largely excitation wavelength independent. Interestingly, a reversible pH-dependent excitation and emission behaviour was observed, with maximum emission occurring at *ca.* pH 7. In addition, nanofiber emission was found to quench in the presence of aqueous solutions of metal cations in a concentration-dependent manner. Single nanofiber optical measurements showed that fiber emission was reasonably stable under continuous excitation and this permitted the demonstration of their use in quenching-based metal ion detection. In this regard, we show that the properties observed for Cdots may be successfully translated to a novel luminescent 1D carbon nanofiber format.

## Materials and methods

### Synthesis of carbon nanofibers

0.38 g citric acid (Sigma Aldrich) was dissolved in 300 μL deionized H_2_O with the aid of sonication (Branson sonic bath). 80 μL polyethyleneimine 800 MW (PEI) (Sigma Aldrich) and was added to the citric acid solution mixed thoroughly. 40 μL of this precursor solution was deposited on a Anodisc 0.2 μm membrane (Whatman) and centrifuged at 500 rpm for 3 min. The membrane was transferred to a 10 mL glass vial containing 2 mL of aerated toluene (Sigma Aldrich) sealed with a silicone septum and PEEK-made cap. The synthesis was performed at 8 bar using an Anton Parr Monowave reactor with an 850 W unpulsed microwave output power. The heating was ramped over 90 s from room temperature 21 °C to 200 °C with a dwell time at 200 °C of 6 min and a cooling (*via* external compressed air) time of 90 s. The temperature was regulated by the power input using an IR thermometer feedback. The membrane was removed from toluene and left in air to dry. Both surfaces were scratched with a blade to remove any material from the surfaces and separate the nanofibers. The membrane was then transferred to 1 mL 3 mol L^−1^ NaOH for 1 h for the membrane to fully dissolve. The suspension was then centrifuged at 11 000 g for 3 min and the supernatant was replaced with fresh deionized H_2_O, this wash was repeated a further three times. The suspension was dialyzed in a 3500 MWCO membrane (Thermo Scientific) in a 1 L deionized H_2_O bath with 10 water changes.

The experimental yield of the carbon nanofibers was calculated to be 10.1 ± 5.7%, to give a final as prepared dispersion of 0.7 + 0.4 mg mL^−1^ (Table S1†). This is based on a theoretical yield determined by the number of pores (1.84 (±0.22) × 10^9^), Fig. S1A† width volume 1.9 (±0.23) × 10^−18^ m^3^, determined using a diameter of 237.3 ± 39.2 nm and a height of 60 μm. The theoretical mass of 8.7 mg was then calculated using the density of amorphous carbon of 2.0 g cm^−3^.

### Metal ion sensing

250 μL of the prepared carbon nanofiber suspension was aliquoted into a quartz cuvette and the emission spectrum was recorded. To this, a volume of metal ion solution was added so that the final concentration was 3.3 × 10^−3^ mol L^−1^. The suspension was lightly mixed and the emission spectrum was recorded again after 1 min. This was repeated for the range of metal ions.

### Fe(ii) Fe(iii) concentration study

250 μL of the prepared carbon fiber suspension was deposited in a well plate and the emission spectra were recorded. To this, a volume of Fe(ii) or Fe(iii) was added to each suspension so that the resulting concentrations were in the range of 6.2 × 10^−6^ to 5 × 10^−3^ mol L^−1^. The suspensions were lightly mixed and the emission spectra were recorded again after 1 min.

## Results and discussion

### Microwave synthesis of luminescent carbon nanofibers

The synthesis of sp^2^ nanomaterials typically requires a high-energy input in the form of elevated temperatures (500–1000 °C) which provide access to gas phase precursors.^4^ This is also true for nanodiamonds, which are typically accessible through detonation techniques. In contrast, carbon dot materials can be prepared using less energy demanding bottom up approaches which typically involves decomposition and subsequent polymerization of carbon precursors at moderate temperatures.^54^ Citric acid is one of the most commonly used sources of carbon due to its low carbonization temperature (<200 °C).^37,55^ The observation of improved optical properties upon N-doping^44^ has resulted in a large number of methods involving the reaction of citric acid with amines,^56–58^ and linear^34^ and branched polyamines.^59^ The preparation of carbon dots by microwave assisted pyrolysis is a very common method of synthesis which involves moderate reaction times and offers the potential for industrial scale up.^42^ This technique has been used to prepare carbon dots from a range of precursors to yield blue to red emitting dots.^53^ The preparation of carbon dots by microwave pyrolysis of citric acid and amines has also been reported.^57,60,61^

In this study we wished to explore whether performing this reaction in a template would provide access to 1D luminescent materials. We previously demonstrated the use of membrane filters to template the synthesis of highly luminescent polymer fibers of uniform morphology.^62^ The use of microwave heating in this way has the advantage of greatly reducing the reaction time and providing a uniform heating, which is important in the synthesis of cylindrical materials to ensure the tips of the fibers are reacting under the same conditions as the body.

**Scheme 1:**
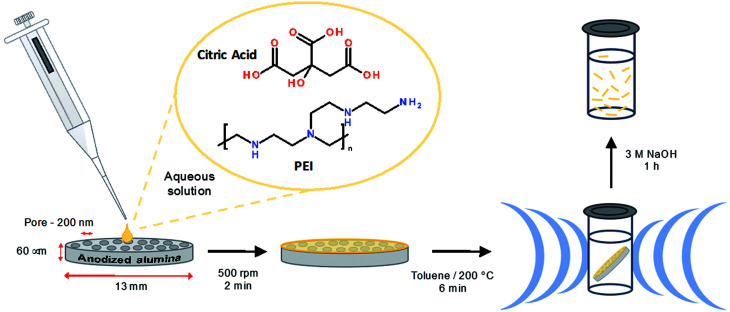
Overview of the synthetic procedure.

Briefly, citric acid and PEI (800 MW) were mixed in water and deposited on a 200 nm nominal pore diameter anodized alumina filter membrane, which was subjected to mild centrifugation (500 rpm) to assist in the loading of the reaction solution. The membrane was then transferred to a glass reaction vial containing 2 mL toluene, which was sealed and subjected to microwave heating at 200 °C under 8 bar pressure for 6 min. Upon completion of the reaction the membrane colour was found to have changed from a white to a yellow appearance (Fig. S2†). The excess material at the surface of membrane was removed using a scalpel and the membrane held material was liberated by dissolving the membrane in NaOH (3 M) followed by dialysis (3500 MW) against water, see Scheme 1. Dialysis was continued until no luminescence was observed in the concentrated solutions of the dialysate (10 × 1 L). This luminescence is attributed to small fragments of broken fibers and partially reacted materials. The separated large molecular weight material remains well-suspended in water for at least 12 h (*ca.* 65% of the material, as measured by optical absorbance, remains in suspension at 12 h; see Fig. S3†) and may be readily re-suspended by gentle agitation.

The experimental yield of the carbon nanofibers was determined to be 10.1 ± 5.7%. This value is based on the theoretical yield obtained using the volume and number of membrane pores. This was calculated using a density of amorphous carbon of 2.0 g cm^−3^. This value is in line with values used previously for low temperature (400–500 K), solvent-based synthesis of carbon dots.^34,63,64^ This yield gave a final as prepared dispersion of 0.7 ± 0.4 mg mL^−1^. This calculation is an estimate and relies on the assumptions that (1) the membrane contains a uniform pore structure and (2) that every pore is fully filled. However, it is not uncommon to have branching and fracturing within pores is and the lower average carbon nanofiber length of 10.5 (±5.3) μm compared to the pore length suggests that there may be partial filling.

### Characterization of carbon nanofibers

**Fig. 1:**
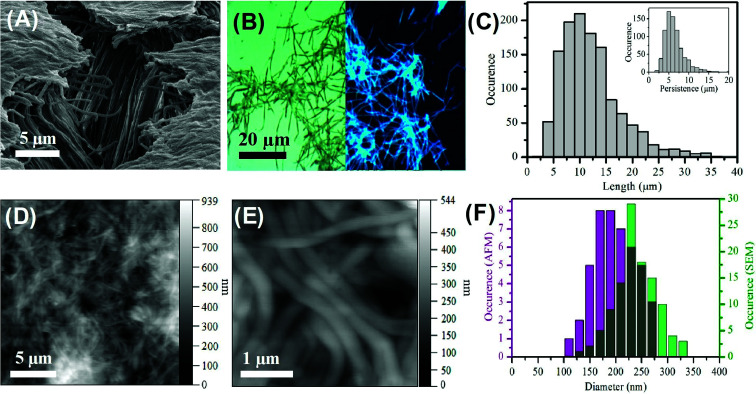
(A) SEM image of aligned carbon nanofibers. (B) Combined epifluorescence (*λ*_ex_ = 390 nm) and bright field images (×100 magnification) of carbon nanofibers. (C) Length distribution of nanofibers collected from bright field imaging, *n* = 1250. Inset – persistence length, *n* = 750. (D) and (E) AFM height images of carbon fibers dried in air. (F) Comparison of the diameter of carbon nanofibers derived from AFM (purple) and SEM (green) data.

The formation of the carbon nanofibers was investigated by scanning electron microscopy (SEM); see Fig. 1A and S4† respectively. The image of the membrane following synthesis and partial digestion reveals a dense forest of aligned nanofibers, which confirms a high yield of nanofiber formation. It is also possible to see individual nanofibers that have become detached. These images compare favourably with those previously reported for the templated synthesis of polymeric nanofibers.^62^ Analysis of SEM images of multiple fibers indicated the Gaussian average diameter of the nanofibers at the fiber mid-point to be 237.7 ± 39.0 nm (*n* = 110); see Fig. 1F. This diameter is somewhat larger than that of the mouth of the membrane pores, which is found to be 212.9 ± 30.6 nm; see Fig. S1.† This difference in size is attributed to the gold sputtering method used to record the images.

The high magnification TEM does not reveal any discernible lattice fringes (Fig. S5A–C†) which indicates the presence of an amorphous carbon structure. This is in agreement with previous TEM measurements on carbon dots prepared from citric acid.^58,65,66^ The uniform composition is further evidenced by the STEM images (Fig S5D–F†) which reveals uniform scattering. At lower magnification some defects in the structure are apparent which suggests the presence of hollow regions (Fig. S5C†). These hollow regions show the amorphous structure extends from the surface to the bulk of the fibers. The clean surface of the carbon fiber is also apparent with an absence of any carbon dot particulate material.

The bright field optical image recorded for carbon nanofibers deposited on a clean glass slide reveals a dense mat of individual fibers with a minimal amount of debris material; see Fig. 1B. This image confirms the presence of discrete, distinct fibers along with some smaller deleterious fragments. Significantly, the corresponding epifluorescence (*λ*_ex_ = 390 nm) image reveals the nanofibers to be highly luminescent. The direct comparison of the bright field and epifluorescence image of the same region of nanofibers shows the entire sample to be luminescent; see Fig. S6.† This luminescence is found to persist along the length of the nanofiber. The epifluorescence image also shows uniformity of fluorescence along the individual nanofibers and no evidence of brighter or darker regions, suggesting homogeneity within the nanofiber. It should also be noted that the small amount of deleterious material observable in the bright field image shows luminescence in the epifluorescence image. This non-fibrous material is attributed to fragments of nanofibers that were generated during the processing, and not to contamination from other sources. The average length of the nanofibers was found to be 10.5 ± 5.3 μm (*n* = 1250) with an average persistence length of 5.7 ± 2.4 μm (*n* = 750); see Fig. 1C. The longest nanofiber observed was found to in the range of 39 μm indicating an aspect ratio of *ca.* 200. The fact that the typical fiber lengths are shorter than the reported thickness of the alumina membrane (approx. 60 μm) is likely due to the scraping process employed to remove excess carbon material from the top and bottom surfaces of the template and to mechanical agitation of the nanofibers during purification and re-suspension.

The carbon nanofibers were also characterized by atomic force microscopy (AFM) in amplitude modulation mode in air on a glass substrate. Dense mats of nanofibers can be seen; Fig. 1D and E. Images recorded following 1 : 2 dilution of the parent nanofiber suspension revealed individual fibers by AFM; Fig. S7.† The mean nanofiber diameter determined from AFM was found to be 206.9 ± 40.5 nm (*n* = 54). This is narrower than the value determined from SEM (see Fig. 1F) and comparable to the size found for the pore openings. AFM probing of the surface of bundles of carbon nanofibers revealed the surface to possess some lateral striation as well as some dents horizontally which could be a result of the internal pore structure or possibly air bubbles creating hollow points in the nanofiber during formation, Fig. S7C and D.† The dimensions of the carbon nanofibers measured by the different microscopy techniques are compared in Table S2.† The close agreement of the carbon nanofiber external dimensions to the nominal internal pore dimensions within the template structure suggests that the precursor solution filled the template pores effectively prior to microwave treatment and that this provided for faithful replication of the pore geometry in the produced nanofibers.

**Fig. 2:**
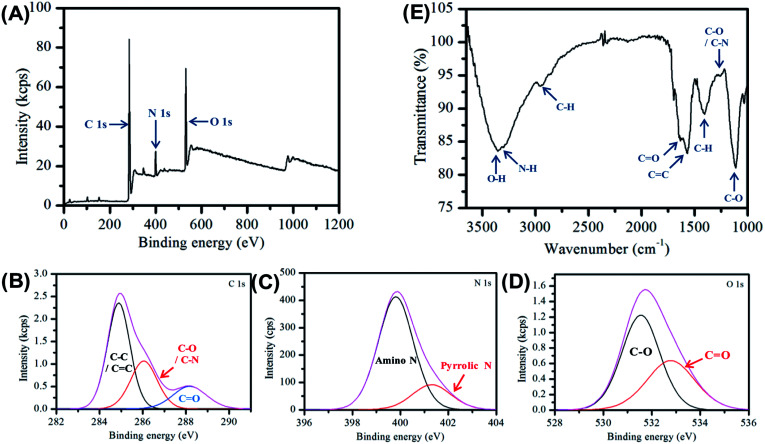
(A) XPS of carbon nanofiber sample deposited from aqueous suspension and dried in air. (B)–(D) High resolution XPS of the relevant C 1s, O 1s and N 1s regions of the XPS spectra (E) FTIR spectra of carbon nanofiber sample in air.

The carbon nanofibers were further characterized by X-ray photoelectron spectroscopy (XPS) and Fourier transform infrared spectroscopy (FTIR) see Fig. 2. The XPS data provides important information on the surface groups in the nanofiber sample and is dominated by three peaks at 286.3 eV, 400.6 eV and 531.9 eV, which are assigned to C 1s, N 1s and O 1s respectively. The detailed assignment of these constituent peaks is given in Table S3.† Three peaks are present in the C 1s spectrum shown in Fig. 2B, which are assigned to C–C/C=C indicating the presence of aliphatic and/or graphitic carbon,^58^ and C–O/C–N, and C=O, respectively, Analysis of the high resolution N 1s spectrum provides further detail of the composition, here two bands are observed due to amino and pyrrolic nitrogen at 400.6 eV and 401.3 eV respectively, see Fig. 2C. In Fig. 2D the O 1s is given, here the C=O and C–O. The elemental composition was determined to be 75% carbon, 17.5% oxygen and 7.5% nitrogen, consistent with previous observations made for Cdots prepared using citric acid and amine-based precursors.^61^

Raman measurements (*λ*_ex_ = 785 nm) did not reveal the presence of D or G bands (Fig. S8†), this is due to the highly amorphous carbon structure of the fibers as seen from the TEM images (Fig. S5†) and is similar to previous observations for highly fluorescent carbon dots prepared by citric acid.^58,67^

FTIR spectroscopy was used to provide additional information on the molecular composition of the carbon nanofibers, Fig. 2E. The spectrum is dominated by a broad feature, which may be ascribed to O–H (3356 cm^−1^) and N–H (3293 cm^−1^) vibrations. The presence of these groups typically results in increased hydrophilicity. The FTIR also shows the emergence of a new peak at 1570 cm^−1^ characteristic of C=C bonding which is an indication of the formation of graphitic material during the carbonization process. In addition, there is a strong band at 1635 cm^−1^ assigned to the stretching vibration of the amide bond C=O.^56^ Full assignments of the FTIR peaks are given in Table S4.† The XPS and FTIR results suggest the presence of a π-conjugation as well as polar groups in the carbon nanofibers formed.

Next, we investigated the optical, *i.e.*, absorption and luminescence, behaviour of the nanofibers, which is a key property of interest. This was investigated for carbon nanofibers either (a) dispersed in solution or (b) deposited on a surface. The UV-visible absorption spectrum recorded for carbon nanofibers dispersed in aqueous suspension shows a strong rising absorbance in the UV and an absorption feature at 350 nm together with a scattering contribution that is apparent across the spectral window. The emission spectrum recorded upon excitation of the sample at 350 nm yields a single band centred at 450 nm, with a relatively narrow FWHM of 73 nm, the bandwidth of which compares well to that of common molecular dyes and quantum dots.^68^ The presence of a significant Stokes shift of 100 nm is an indication of internal energy loss in the absorption/emission process. The excitation spectrum obtained by probing the emission reveals a structured spectrum with two bands present at *ca.* 250 nm and 350 nm, respectively.

**Fig. 3:**
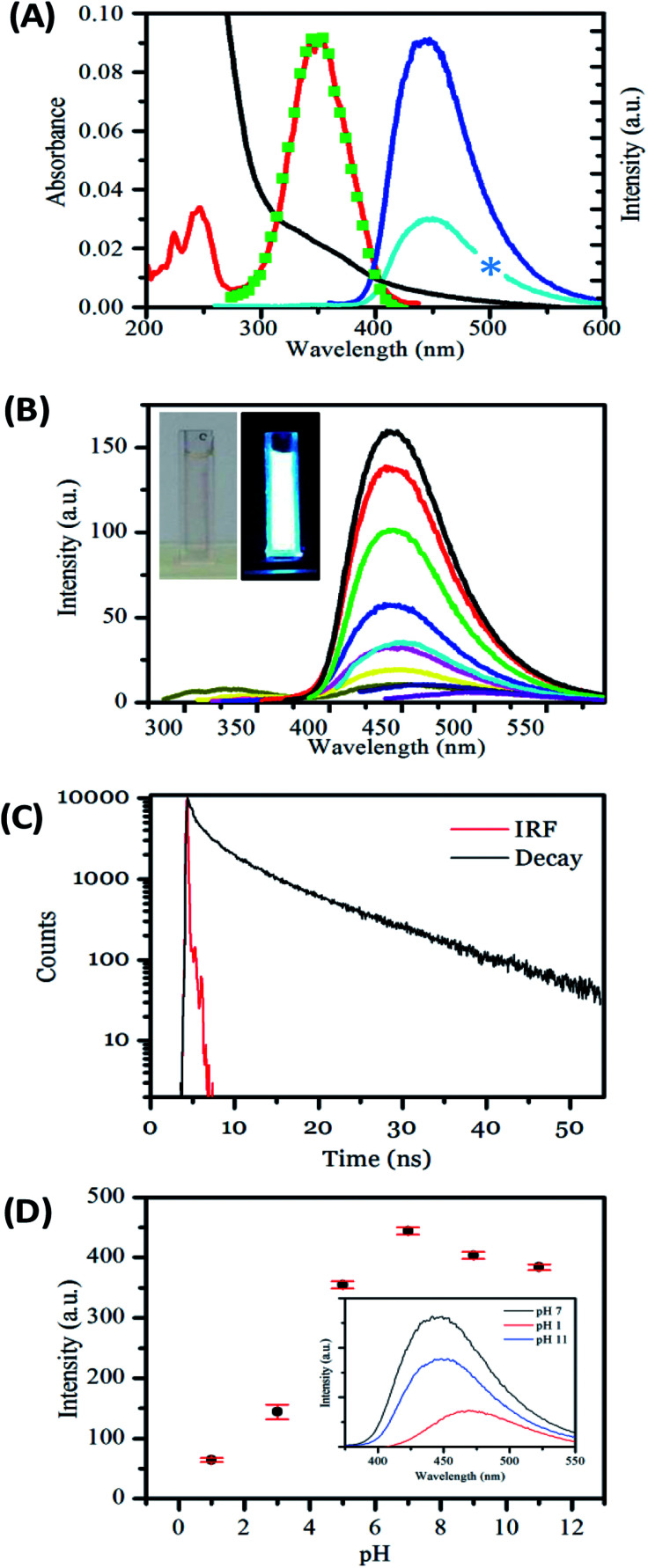
(A) Absorbance (black), excitation for 450 nm emission (red) emission for 350 nm excitation (dark blue), emission for 250 nm excitation (light blue), excitation reconstructed from wavelength dependent emission (green), spectra of an aqueous suspension of carbon nanofibers. Blue star represents deleted data corresponding to the second harmonic of the light blue emission spectrum. (B) Emission spectra taken at 20 nm incremental excitations from 290 nm (blue line) to 450 nm (purple line) showing little excitation dependent shift. Inset shows photographs of a sample in the cuvette the measurements were recorded in natural light and under 365 nm UV lamp. (C) Lifetime of carbon nanofiber suspension, in aqueous solution at pH 6.8. (D) Graph showing the effect of pH on emission at 350 nm excitation and (inset) its reversibility.

The carbon nanofiber emission is found to be largely independent of the excitation wavelength with a single emission band observed when the sample is excited at wavelengths between 220 nm and 400 nm; see Fig. 3B, S9 and S10.† Though at excitation wavelengths greater than 410 nm a small amount of red-shifted emission is detectable, this emission is only a minor contribution. The bright luminescence of the suspension can be seen in the photograph of a cuvette illuminated at 365 nm, and may be compared with the clear/yellow suspension as it appears in natural light; see Fig. 3B, inset. Monitoring of the luminescence lifetime reveals a biexponential process where the majority of the decay occurs with a lifetime of 1.02 ns (69%) with a minor component of 9.01 ns (31%) also being detected; see Fig. 3C and Table S5.† The quantum yield of nanofiber emission was also measured to be 4.8%. The luminescent behaviour of the carbon nanofibers exhibits some similarities and differences to the related Cdot systems. The emission peak at *ca.* 450 nm and Stokes shift of 100 nm is very similar to the values obtained for Cdots prepared by microwave treatments of the precursors used in this study (445 nm/95 nm)^69^ and the general observations for citric acid sourced carbon dots, prepared by microwave^57,69^ and hydrothermal^58,65,67,70–72^ methods (*λ*_em_ = 425–460 nm per stokes shifts 75–110 nm). In addition, the fluorescence lifetime of the luminescent carbon nanofibers is largely similar to that of the citric acid based carbon dots, which have been reported to exhibit biexponential decays comprising sub ns, and 3–15 ns components (Table S6†). However, the quantum yield of 4.8% for luminescent carbon fibers is low compared to values reported for carbon dots prepared using similar precursors.^34,57^ (Table S6†). The moderate quantum yield observed for the nanofibers may be due to the presence of a larger number of defects that give rise to non-radiative relaxation of excited states or due to some reabsorption of the emitted light.

To understand the origin of these spectral bands it is useful to consider related Cdot systems. A clear picture of the chemical origin of the photoluminescence in Cdots is yet to emerge and continues to be a subject of debate and scrutiny.^71,73–76^ The origin of the excitation and emission bands in Cdots is quite complex. In general, the luminescence of Cdots has been attributed to (i) quantum confinement effect or conjugated π-domains of the sp^2^ carbon core, (ii) functional group rich surface domains and (iii) molecular based emission.^32^ The presence of the two band structures at *ca.* 250 nm and 350 nm in the excitation spectrum has previously been attributed to the contribution of more than one excited state to the emission. The higher energy transition (250 nm) has been previously attributed to π–π* transitions in the sp^2^ network of the carbon core while the lower energy, more intense band at 350 nm is characteristic of n–π* transitions of N and O containing surface groups.^76^

The fluorescence of carbon dots has been described as a unique cocktail of polyaromatic hydrocarbons, with importance given to the ratio of sp^2^ to sp^3^ carbons.^74^ However, while XPS data reveals the presence of sp^2^ and sp^3^ carbon in the luminescent nanofibers the absence of lattice structures in the TEM reveals that they do not occupy distinct core, and surface regions as previously described by Xu et. *al.*^77^ Thus, their role in the emission of the nanofibers is difficult to elucidate.

Cdots commonly display multiple emissive states^56,71^ a phenomenon that is related to the reaction conditions, including temperature as well as the specific precursors, employed. The presence of a short 0.96 ns emission decay together with a longer 7.8 ns component may be explained by a distribution of deactivation pathways within the nanofibers arising from the presence of competing pathways within nanofibers or the presence of different pathways due to differences between nanofibers.^73^ The short lived nature of the emission suggests that there may be ultrafast deactivation processes at play, which have been previously observed for Cdots.^73^ However, the observation of predominantly single wavelength emission here in this work is quite consistent with the presence of a single emitting pathway and a homogeneous sample. Excitation independent blue emission has been observed for Cdots prepared by the reaction of citric acid and amine precursors, and has been explained by invoking a molecular origin for the emission based on, *e.g.*, citrazinic acids.^36,56,72,75^ The excitation/emission behaviour reported in the work by Song et *al.* is very similar to that observed in this work suggesting that the emission from the carbon nanofibers may also be related to the presence of citrazinic acid-type moieties.^36^ Importantly, the contribution of such molecular species is most significant for hydrothermal processes in which have been prepared at less than 300 °C. These preparation conditions are similar to those employed for nanofibers in this work. The similarity in the composition and spectral properties of the nanofibers to their related Cdots allows us to propose a similar mechanism of formation. This involves the dehydration of the citric acid carboxylic groups under high temperatures, here 200 °C, followed by the polymerization of the resulting carbon fragments to form a core. The presence of the amine-containing polyethyleneimine is expected to result in the incorporation of pyrrolic nitrogen within citrazinc type surface components.

Carbon dots formed from the reaction of citric acid and amines have shown pH responsive luminescence.^40,56,75,78,79^ Next, the sensitivity of the carbon nanofiber luminescence to the solution pH was investigated. The emission was found to be sensitive to changes across the pH range 1 to 12; see Fig. 3D. The nanofibers are highly emissive from weakly acidic pH to alkaline pH with the greatest intensity found at pH 7. However, in line with previous observations for carbon dots, the emission intensity is significantly reduced under acidic conditions (pH < 5).^40,56,75,78,79^ The position of the emission maximum is also found to slightly shift as a function of pH. As the pH is moved from alkaline to acidic conditions, the emission *λ*_max_ is found to redshift from 447 nm at pH 12 to 471 nm at pH 1, with a corresponding shift in the excitation spectra; see Fig. S11, and Table S7.† This pH dependent excitation/emission behaviour is also found to be reversible; see Fig. 3D, inset. These observations concur with those previously reported for Cdots prepared using citric acid and amine precursors and attributed to protonation and deprotonation of surface groups. In this context, the loss of fluorescence of the nanofibers under highly acidic pH can be considered to be a consequence of increased electron withdrawing efficiency of the –COOH group compared to the –COO^−^ group.^56,78^ These observations confirm the important role of surface functionality in the luminescence behaviour of the nanofibers. The fact that the carbon nanofibers show maximum emission under conditions of physiological pH suggests their potential utility as environment responsive materials in fluorescence-based sensing and imaging applications.

### Application of luminescent carbon nanofibers for metal ion sensing

The application of Cdots for metal ion sensing has attracted significant attention, with quenching observed in the presence of a number of metal ions.^5,58,60,78,80–85^ Nitrogen doped Cdots prepared from citric acid have displayed selectivity to different metal ions, including Fe(ii), Fe(iii), Cu(ii) and Co(ii), see Table S9.† Sensitive and selective detection of Cu(ii) by polyamine coated Cdots was attributed to an inner filter effect arising from the UV-visible absorption of the cupric amine species formed at the surface. While quenching due to electron transfer has been reported for a number of ions.^58,81,82^ The influence of surface states on the luminescent properties of Cdots has previously been exploited for metal ion sensing.^58^ In the case of Fe(iii), it has been postulated that binding to surface phenolic hydroxyl groups leads to electron-transfer from the Cdot excited-state to the d orbital of the Fe(iii) ion, which results in quenching.^58,83^ The greater stability constant for Hg(ii) and carboxylic acid groups compared to other metal ions has also given rise to selective detection.^81^

**Fig. 4:**
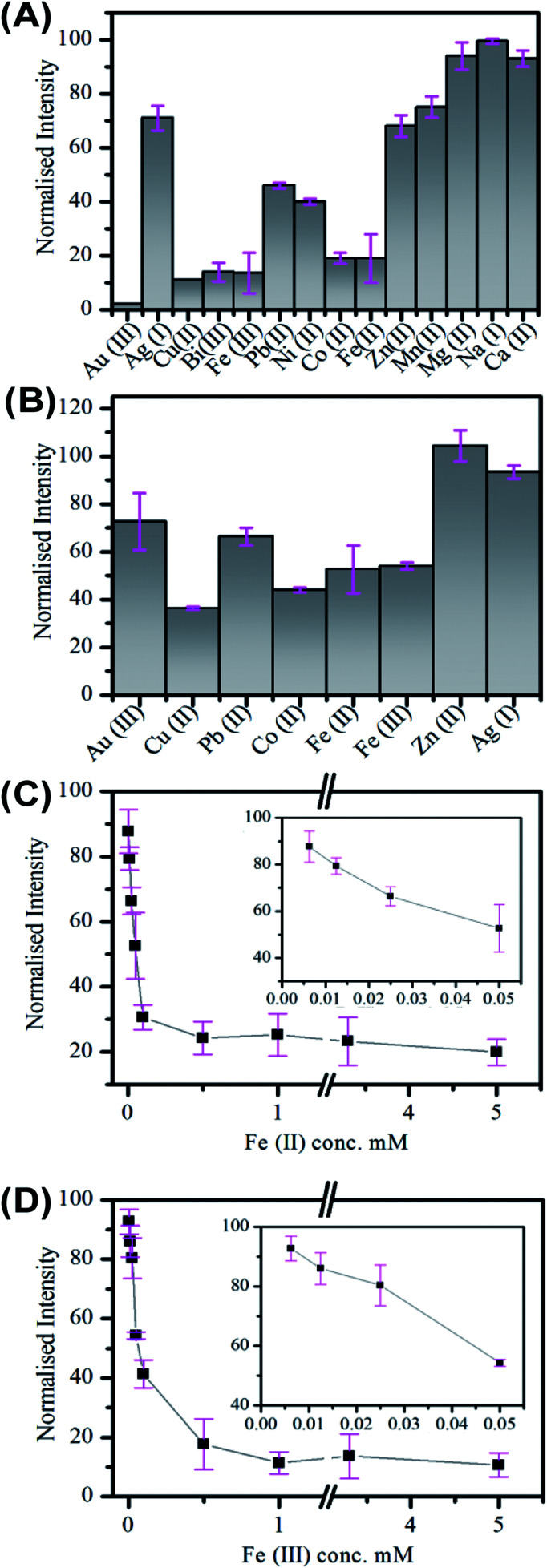
Emission quenching of carbon nanofibers in aqueous suspension in the presence of metal ions at (A) 3.3 mM and (B) 50.0 μM concentration. (C) and (D) The normalised emission of carbon fibers in the presence of Fe(ii) and Fe(iii) respectively as a function of concentration from 6.25 μM to 5.0 mM. Inset shows in greater detail the low concentration region from 6.25 μM to 50.0 μM. All emission was taken at 350 nm excitation.

The response of the carbon nanofiber luminescence to the presence of (a) 3.3 mM and (b) 50 μM concentrations of the biologically and environmentally relevant ions; Au(iii), Ag(i), Pb(ii), Co(ii), Cu(ii), Fe(ii), Fe(iii) and Zn(ii) was investigated. These measurements were performed in a low volume cuvette containing a 500 μL nanofiber dispersion of 0.36 mg mL^−1^, comprising approximately 2.3 (±1.3) × 10^8^ nanofibers. The extent of quenching observed in the presence of 3.3 mM ion concentration was found to vary, with the greatest quenching observed for Co(ii), Cu(ii), Fe(iii) and Fe(ii), see Fig. 4A. The quenching behaviour was also examined at lower (50 μM) ion concentrations; see Fig. 4B. Again, the greatest sensitivity is seen for Co(ii), Cu(ii), Fe(iii) and Fe(ii) ions. Fig. 4C and D shows the change in intensity observed upon addition of Fe(ii) and Fe(iii) metal ions. These ions exhibit similar behaviour and the intensity is found to plateau at ∼50 μM ion concentration with ∼80% quenching. The incomplete quenching suggests that there are a number of luminescent sites which are inaccessible to the metal ions. These may either may originate in the core of the nanofiber (consistent with the pH dependent emission observations; see Fig. 3D) or they are being blocked by defects in the surface meaning they cannot reach the luminescent surface site.

The selective detection of metal ions is challenging, especially as the origin of the quenching interactions of Cdots is unproven and many theories, including static quenching, dynamic quenching FRET and PET, have been used to explain these processes.^85–87^ In the case of the carbon fibers the quenching by Co(ii), Cu(ii), Fe(ii) and Fe(iii) may indicate interaction with oxygen rich species. While the lack of significant quenching in the case of Zn(ii) may be due to interaction with surface amine groups, which has previously been observed to results in enhancement of Cdot emission.^78^ The situation becomes complicated for Au(iii) and Ag(i) as these metals may be reduced at the Cdot surface. This has been previously observed for Ag(i) and resulted in enhancement of emission.^88^

The quenching in the presence of the wide range of ion types suggests that the metal ions coordinate through nonspecific interactions with surface groups.^58^ This presents a barrier to the application of these materials in competitive media. Overcoming this challenge will require further optimisation of the nanofiber surface. As highlighted in the review by Roy *et al.* this may be achieved by (i) changing the precursor and (ii) removing surface molecular inhomogeneity and morphologies.^89^ In the case of (ii) we believe this may be achieved through subsequent annealing and/or surface functionalisation.

### Spectroscopic study of individual luminescent carbon nanofibers

**Fig. 5:**
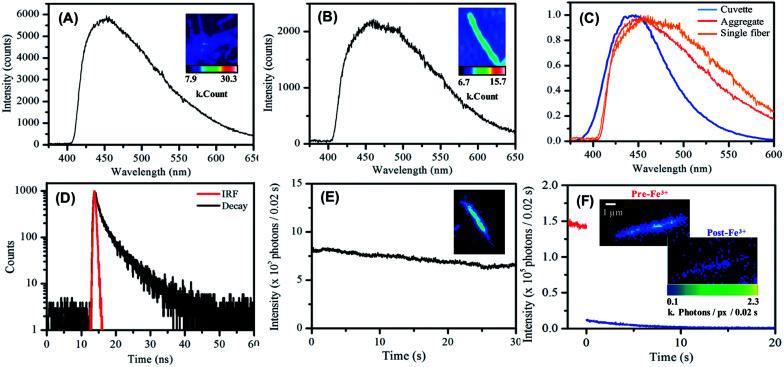
(A) Emission spectrum of a deposited aggregated nanofibers on glass in air. (B) Emission spectrum of a single nanofiber deposited on glass in air. (C) Comparative emission spectra of the carbon nanofibers in aqueous suspension (blue), deposited and aggregated (red) and deposited single fibers (orange). (D) Lifetime decay for a deposited carbon nanofiber sample on glass in air. (E) Single nanofiber photostability in air over the course of 30 seconds. (F) Single nanofiber photostability in the presence of deionised H_2_O (red) and Fe(iii) solution (blue). Images show first frame of each set of data.

Lastly, the luminescent behaviour of the carbon single nanofibers, deposited from suspension onto glass substrates and dried in air, was investigated.^90^ A representative emission spectrum, with accompanying false-colour photoluminescence emission image, recorded for such a drop-deposited aggregate of carbon nanofibers is shown in Fig. 5A. The overall profile of the emission spectrum is similar to that measured for nanofibers suspended in water. The emission maximum for the dried nanofibers was typically found at *ca.* 450 nm. The slight red-shift in emission maximum compared to that observed for the suspension is likely due to a spectral intensity subtraction by the long-pass filter used in the fluorescence microscope collection optics. The relatively greater contribution of low-energy emission to the overall nanofiber mat emission spectrum appeared to be notable at longer wavelengths.

Single nanofiber emission spectrum and image data was also acquired, see Fig. 5B, typical single nanofiber emission spectra exhibited further reweighting of emission intensity with the low energy side of the emission spectrum being yet more pronounced with respect to the high energy side Fig. 5C.^90^ Photoluminescence decay data were measured for dried nanofiber mats in air using the time-correlated single photon counting (TCSPC) technique (*λ*_ex_ = 400 nm). Similar to nanofiber suspensions, a double exponential function was used to fit the decay data. The shorter lifetime component was *ca.* 1.4 ns (76%) and the longer component was *ca.* 5.3 ns (24%), see Fig. 5D and Table S8.†

Single nanofiber emission photobleaching measurements were also undertaken by acquiring wide-field emission image stacks, comprising consecutively measured 0.02 s frames, of emission from individual nanofibers under continuous excitation at 404 nm and extracting a value of the per-pixel emission intensity for a nanofiber as a function of time; see Fig. 5E and S15.†^91^ The nanofibers exhibited promising photostability with a loss *ca.* 25% emission intensity over 30 s of steady illumination in air. This approach to the real-time, *in situ* monitoring of nanofiber emission intensity was also employed to record the response of single nanofiber emission upon exposure of a nanofiber to an aqueous solution of Fe(iii) ion; see Fig. 5F and S16.† In this regard, significant (>90%) and prompt (within 10 s) quenching of emission intensity was typically observed. This significant response observed for individual carbon nanofibers suggests the potential applicability of single fibers for nanoscale sensing and detection applications.

## Conclusions

In the short time since the first reported synthesis of luminescent Cdots, interest in their preparation and application has grown tremendously. In this work we have demonstrated the ability to adapt the preparation of Cdots from citric acid to prepare of blue emitting amorphous nanocarbon fibers (diameter ∼ 200 nm, length ∼ 10 μm) by template-assisted microwave synthesis. The comprehensive characterisation of these fibers reveals several similarities to the properties of the parent Cdots, which includes metal ion quenching and reversible pH-dependent the emission at *ca.* 450 nm, with maximum emission at pH 7. Going forward, the opportunity now exists to exploit the extensive Cdot body of research to provide access to new luminescent materials. Challenges to realising the potential of these materials, such as improving the yield, tuning the emission and improved selectivity in sensing, are the subject of ongoing research in our laboratory. In the specific case of selective sensing we believe this may be achieved through surface modification by chemical functionalisation or annealing. In summary, the introduction of these new 1D luminescent carbon nanofibers offers the exciting potential to further harness the recent developments in Cdot synthesis and applications in the areas of imaging and sensing.

## Conflicts of interest

There are no conflicts to declare.

## Supplementary Material

RA-008-C7RA13383A-s001
