# Supplementary material for: Templated microwave synthesis of luminescent carbon nanofibers
Source: RSC Adv. 2018 Apr 6;8(23):12907–17. doi: 10.1039/c7ra13383a (PMC9079752; doi:10.1039/c7ra13383a)
Supplement: RA-008-C7RA13383A-s001 [file RA-008-C7RA13383A-s001.pdf]

Electronic Supplementary Material (ESI) for RSC Advances.
This journal is © The Royal Society of Chemistry 2018



# SUPPLEMENTARY INFORMATION

# Templated microwave synthesis of luminescent carbon nanofibers

*Clara Deeney[a] Suxiao Wang,[a,d] Samir A. Belhout,[a] Aoife Gowen,[b] Brian J. Rodriguez,[c] Gareth Redmond[a]* and Susan J. Quinn[a]**

[a] School of Chemistry, University College Dublin, Dublin 4, Ireland.

[b] School of Biosystems and Food Engineering, University College Dublin, Dublin 4, Ireland.

[c] School of Physics, University College Dublin, Dublin 4, Ireland.

[d] School of Chemistry and Materials Science, Hubei University, Wuhan 430062, China

Email: gareth.redmond@ucd.ie, susan.quinn@ucd.ie

## MATERIALS AND METHODS

**Synthesis can be found in main paper.**

**Suspension measurements.**

**Absorbance and emission, and fluorescence lifetime analysis.**

The as prepared aqueous carbon nanofiber aqueous suspension was measured in a low volume (500 μL) quartz cuvette. Slit width for excitation and emission data was 5 mm and 5 mm. Absorbance was measured on a Cary 60 and excitation and emission were measured on a Cary Eclipse.

**Fluorescence lifetime measurements.**

The as prepared aqueous carbon nanofiber aqueous suspension was measured in a low volume (500 μL) quartz cuvette. Excitation was 400 nm, collection wavelength was 450 nm. The Instrument Response Frequency was found using Ludox colloidal silica in water.

**Quantum yield measurements.**

The as prepared aqueous carbon nanofiber aqueous suspension was measured in a 3 mL quartz cuvette. The baseline was take with deionized $H_2O$ and an integration sphere was used to collect the absorbance and emission counts.

**pH sensing.**

Emission spectra for suspensions of carbon fibers (absorbance at 350 nm at 0.1) were taken. These carbon fiber suspensions were spun down via centrifugation and resuspended in buffers of known pH (pH 1, pH 3, pH 5, pH 7, pH 9 and pH 11), and the emission spectra was repeated.

For emission switching, the emission spectrum was taken a suspension of carbon fibers in water (pH7). Conc. HCl was added resulting in pH of one (emission spectrum was taken) followed by addition of conc. NaOH until the pH had reached 11 (emission spectrum was taken).

**Metal ion sensing.**

Metal ion sensing measurements are described in the main paper.

**Deposited measurements**

**Bright field/ epifluorecence microscopy imaging.**

A glass microscope slide was soaked in methanol for 1 h and rinsed in ethanol followed by deionized $H_2O$ at least three times and dried under a stream of nitrogen to clean. 10 μL of the prepared fiber sample was deposited on this cleaned slide and left to dry in air. Epifluorescent images were taken at 390 nm, with a 10 s integration time on an Olympus BX51 microscope system.

**AFM.**

A glass microscope slide was soaked in methanol for 1 h and rinsed in ethanol followed by deionized $H_2O$ at least three times and dried under a stream of nitrogen to clean. 30 μL of the aqueous sample was dropped onto the

glass slide and allowed to dry at room temperature. AFM images were taken in amplitude modulation mode in air using an Asylum Research MFP-3D with a PPP-NCH tip (Nanosensors, Switzerland). The instrument was Asylum Research MFP-3D Classic AFM, at a scan rate of 1 Hz.

**Raman.**

The Raman study was performed on a SENTERRA dispersive Raman microscope (Brunker optics) with a 785 nm laser. Settings applied were 1 mW power, 10 second collection times and 10 accumulations. The sample was deposited from aqueous suspension onto a cleaned $CaF_2$ plate, a total of 6 mL as prepared nanofiber was dropped sequentially onto the same area in 10 μL aliquots

**XPS.**

The sample holder was sonicated in ethanol for 30 min, and dried in air to clean. 10 μL of the carbon fiber suspension at a time were deposited into the middle of the sample holder and dried in air at 60 °C. This was repeated until a total of 3 mL was deposited and dried to ensure no gaps between the fibers, revealing the sample holder.

**FTIR.**

A $CaF_2$ plate was washed in ethanol and dried in air and 10 μL of the prepared fiber suspension was deposited on this and left to dry in air. IR images were acquired using a Nicolet iN10 MX Imaging system by Thermo Scientific (Madison, WI, USA). The detector used was an MCTA point array detector, which was liquid Nitrogen cooled. Regions of the IR image corresponding to the fibers were selected by thresholding a principal component scores image that segregated them from the $CaF_2$ slide background. The mean spectrum of the fiber regions was then calculated.

**SEM.**

Once the membrane was removed from the microwave and dried in air, the 'top side' was identified (more uniform pore distribution in anodizing process – appears shinier) and this side was scratched only. The underside was stuck to an adhesive carbon pad and approx. 100 μL 3 mol $L^{-1}$ NaOH was dropped onto the membrane and left for 25 min. The membrane (still attached to the pad) was then rinsed with clean deionized $H_2O$ and left to dry. The carbon pad was then stuck to a sample holder.

Samples were sputter coated with gold, using a Emitech K575X Sputter CoatingUnit, to prevent surface charging by the electron beam. Images were taken using a FEI Quanta 3D FEG DualBeam (FEI Ltd,Hillsboro, USA).

**TEM/STEM.**

Transmission Electron Microscope (TEM) and Scanning Transmission Electron Microscope (STEM) images were recorded on a FEI TITAN TEM/STEM at acceleration voltage 300 kV. The nanofibers were deposited from aqueous suspension onto a holey carbon substrate and dried under a stream of $N_2$.

**Deposited lifetime measurements.**

A glass microscope slide was soaked in methanol for 1 h and rinsed in ethanol followed by deionized $H_2O$ at least three times and dried under a stream of nitrogen to clean. 10 μL of the prepared carbon nanofiber sample was deposited on this cleaned slide and left to dry in air.

Lifetime measurements were taken on a constructed system with 405 nm excitation laser, and collection at all wavelengths. Lifetime was taken to 1000 counts over various regions of the carbon nanofiber sample and the results presented here are the average of all of these measurements. Instrument Response Frequency was Ludox colloidal silica in water.

**Single fiber ion sensing.**

Single nanowire photoluminescence imaging was undertaken using a home-built wide-field epi-fluorescence microscopy system consisting of inverted microscope (IX71®, Olympus, Inc.) equipped with a 404 nm diode laser (iBeamsmart 405-S, TOPTICA Photonics AG), an electron multiplying charge-coupled device (EMCCD) camera (Evolve™ 512, Photometrics), and a 100 × oil immersion objective lens (1.45 NA; UPLSAPO 100XO, Olympus, Inc.) in combination with a 1.6 × tube lens. Samples were prepared by drop casting the carbon nanofiber suspension in uncoated, sterile, 96 well µ-plate (ibidi GmbH). To acquire photoluminescence images, each sample was illuminated at 404 nm at an excitation intensity of 42.8 W $cm^{-2}$ and 100 / 1,000 frames for imaging were acquired with an exposure time of 20 ms at an electron multiplication gain of 200. 50 µL of DI water was first added in to the sample well and 100 frames imaging were acquired after. Then another 50 µL of Fe(III) ion solution was added into the same sample well and 1,000 frames images were acquired. Approximately 10 s in between every two imaging acquirements without under any light exposure. Metamorph® Microscopy Automation & Image Analysis Software (Molecular Devices, Inc.) was used to control the imaging system. Acquired image stacks were subsequently analyzed using Image-Pro® Plus (Media Cybernetics, Inc.), and plotted using Origin® 8.1 (OriginLab Corp.).

## Figures

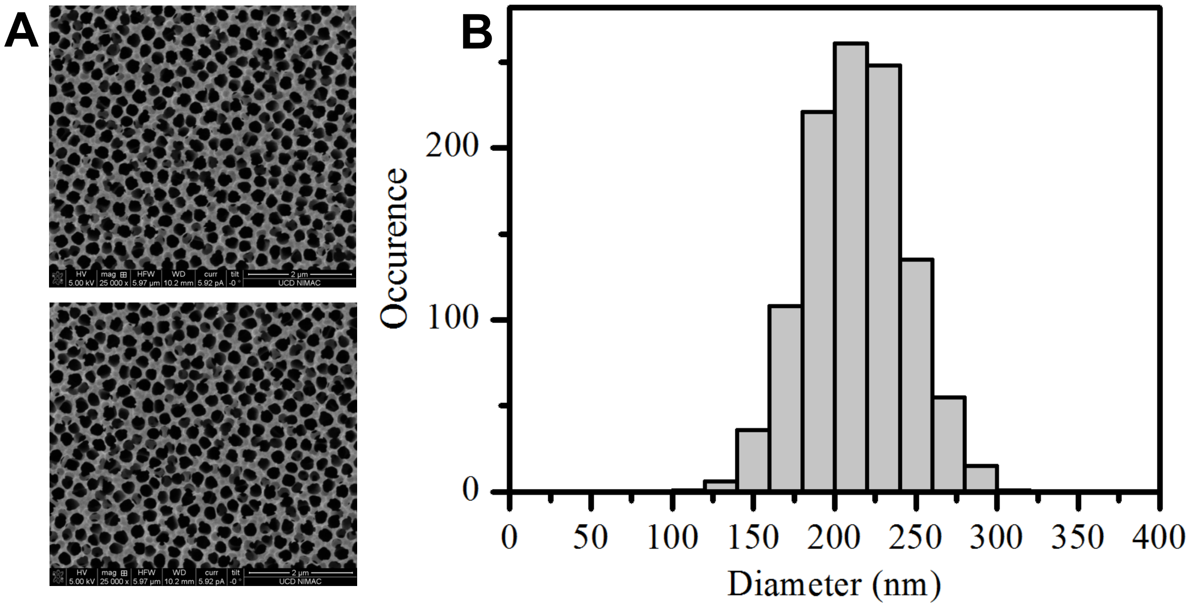

**Figure S1 A** SEM images of empty anodized alumina templates as used in the synthesis of carbon fibers. **B** Size distribution of the diameter of the pore size determined from the SEM images, *n* = 1087.

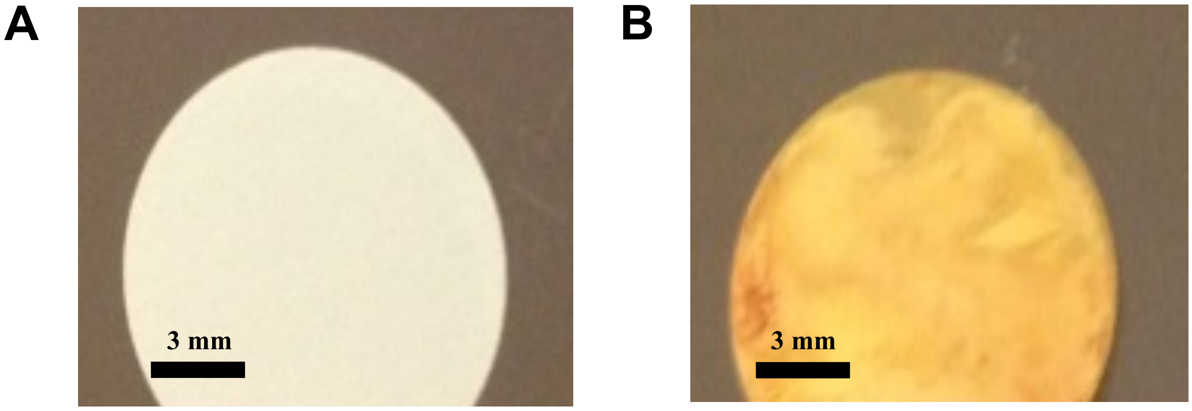


**Figure S2 A** Appearance of membrane prior to microwave reaction. **B** Appearance of membrane following microwave reaction.

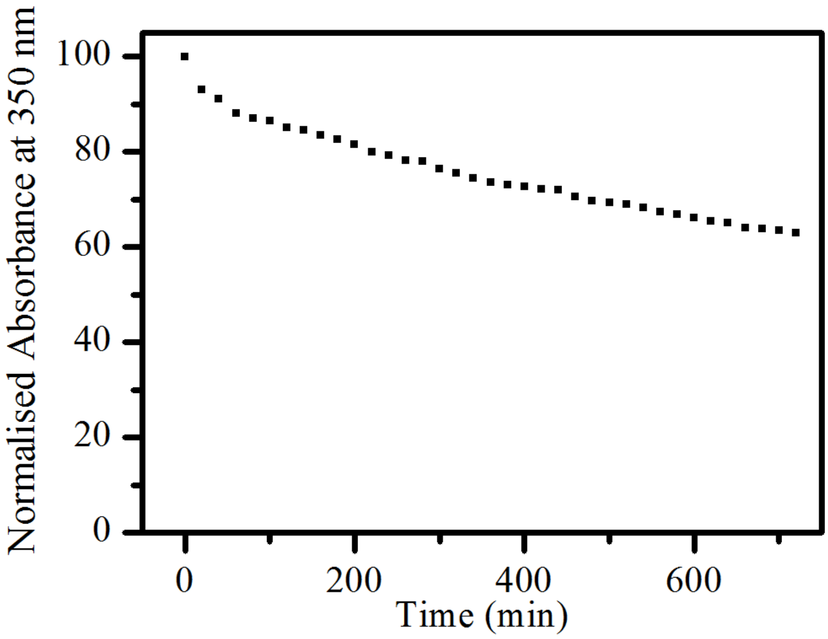


**Figure S.3** Time dependent absorbance (350 nm) of a carbon nanofiber suspension in aqueous solution at 20 °C recorded at 20 min intervals over 12 h.

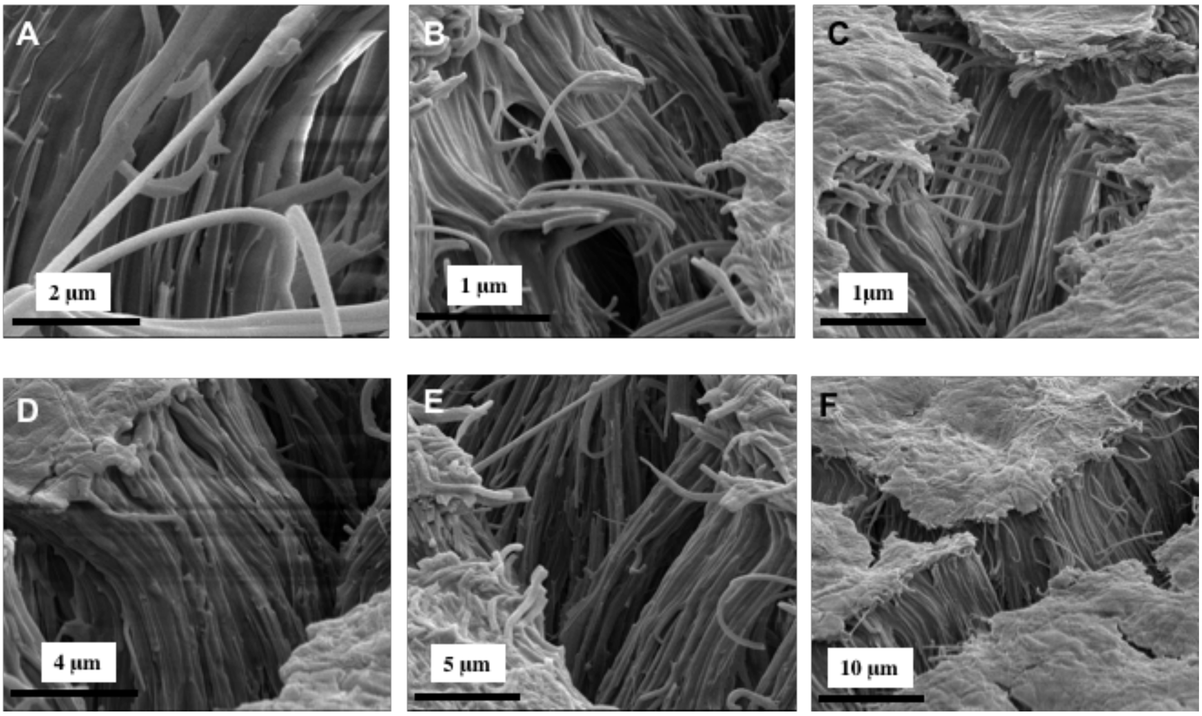


**Figure S4 A-F** SEM of various regions of a membrane following microwave reaction and digestion, showing close packing of multiple fibers in all areas.

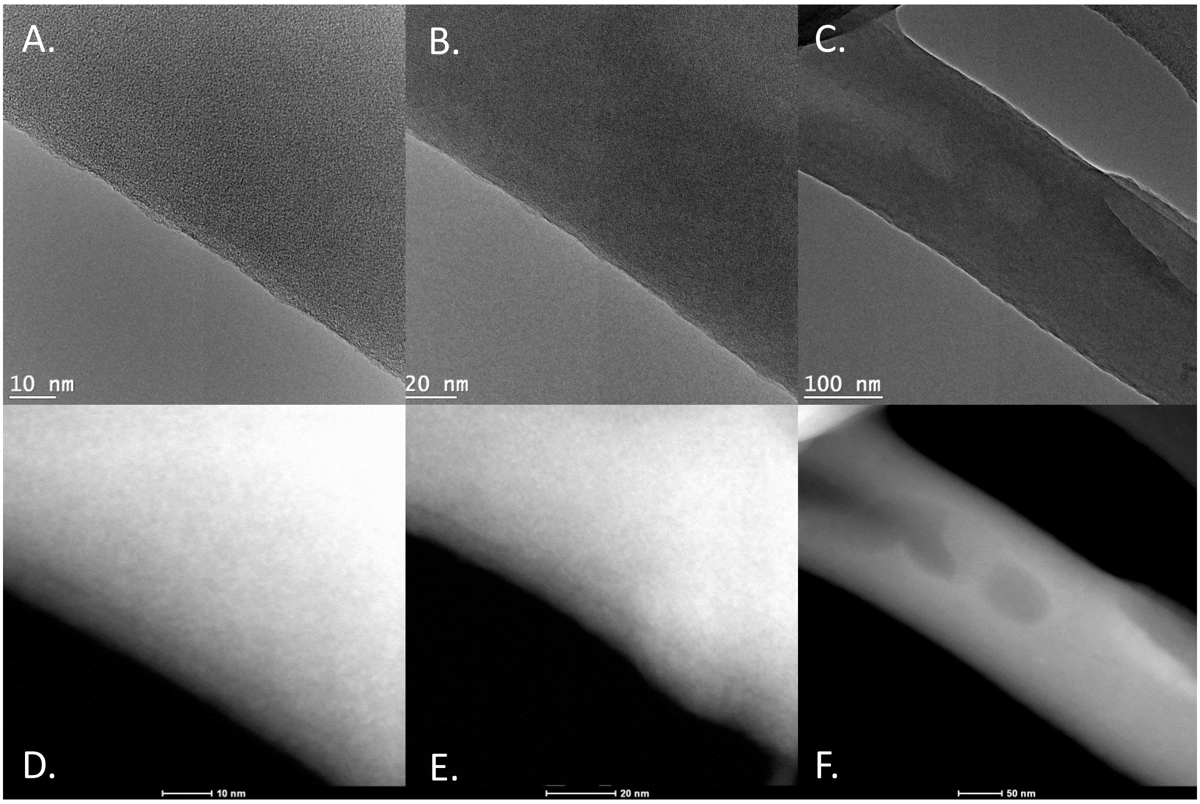


**Figure S5. A-C.** TEM images at various magnifications of the edge region of a luminescent carbon fiber deposited on holey carbon. **D-F** STEM images at various magnifications of the edge region of a luminescent carbon fiber deposited on holey carbon.

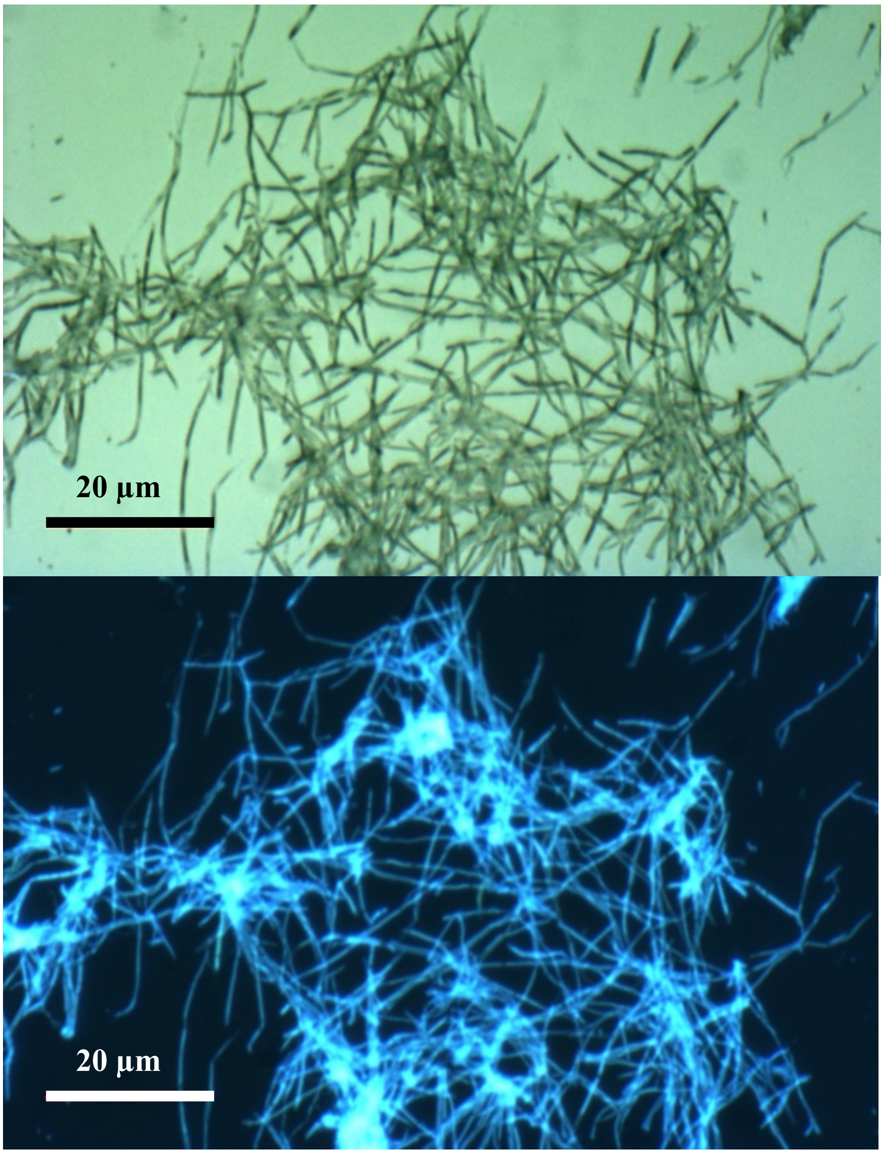


**Figure S.6** Complementary epifluorescent ($\lambda_{ex}$= 390 nm) and bright field images (×100 magnification) of carbon fibers.

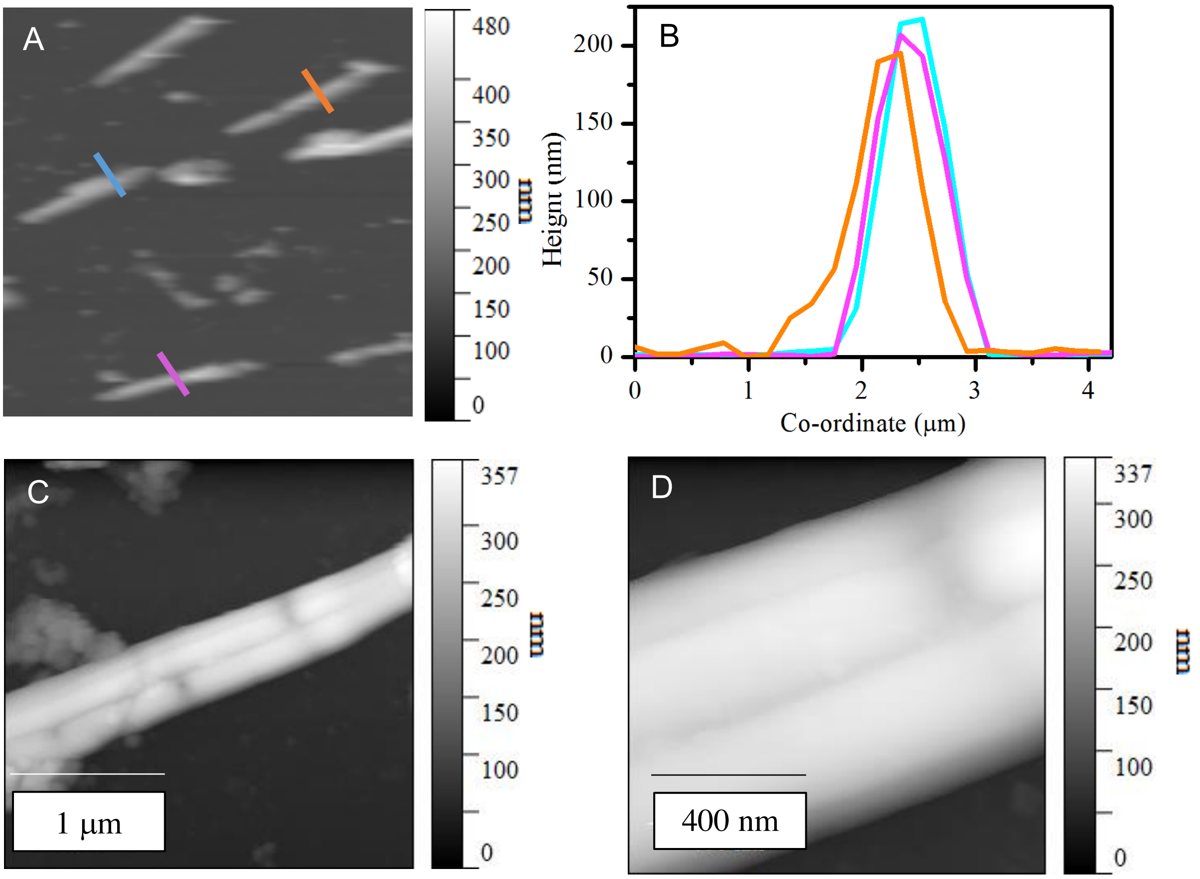


**Figure S7. A** Example of the AFM height trace, at lower concentration of carbon fibers. **B** Line profiles extracted from AFM height traces used to determine fiber diameter distribution. **C D** Zoom image of undivided fibers showing the surface morphology

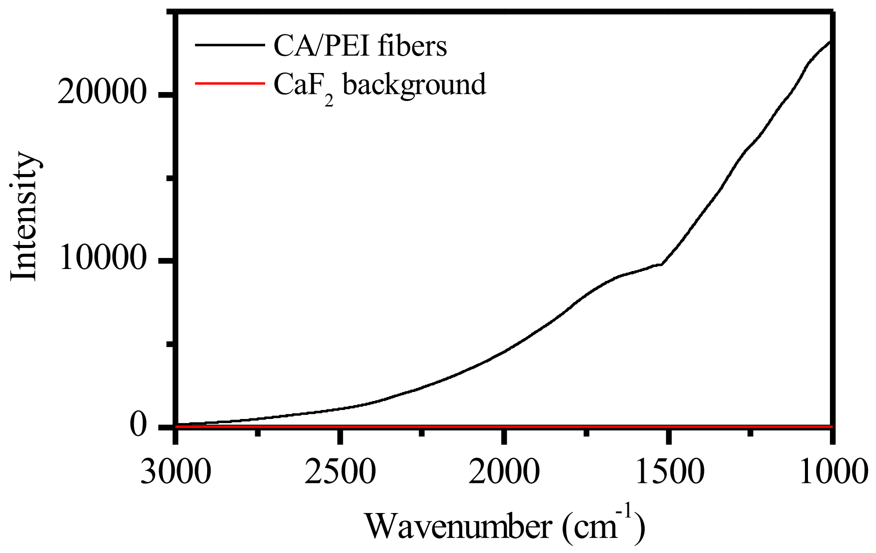


**Figure S.8** Raman spectra of luminescent carbon nanofibers deposited from aqueous suspension onto a $CaF_2$ substrate compared to the $CaF_2$ substrate background. Recorded at 1 mW with 785 nm source.

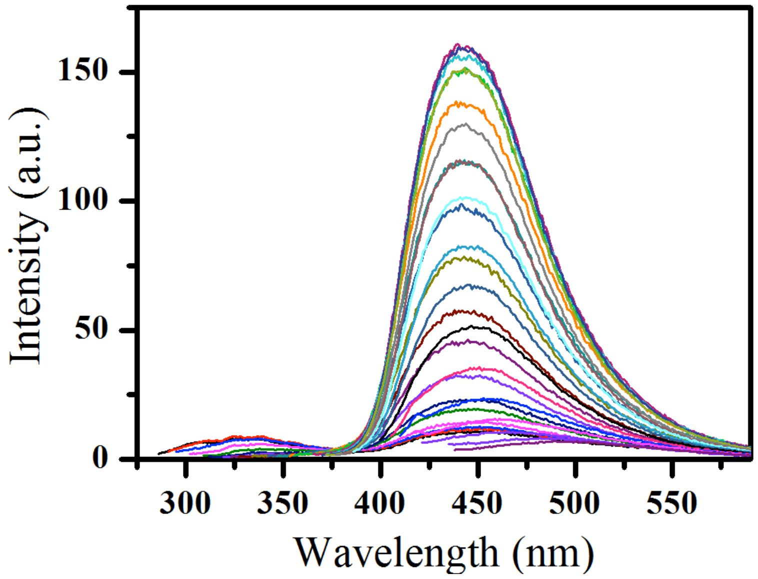


**Figure S.9** Excitation dependent emission at 5 nm intervals from 290 nm to 450 nm in aqueous suspension in quartz cuvette.

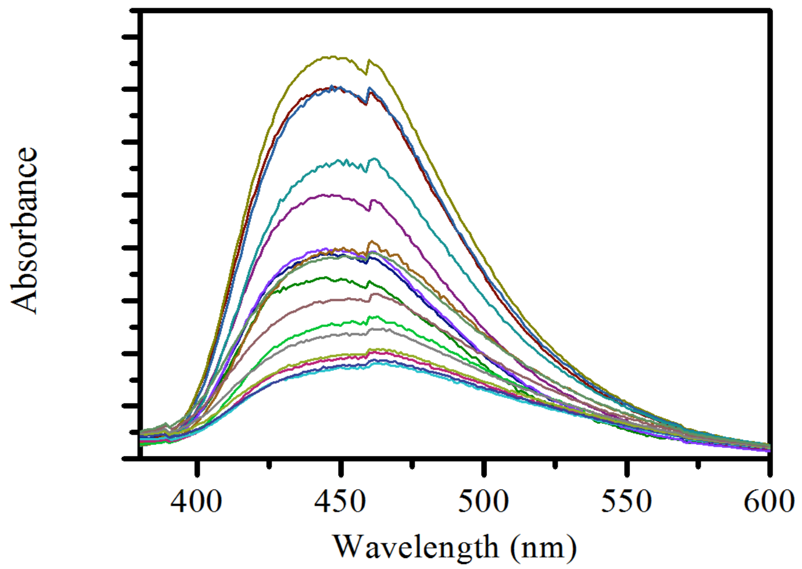


**Figure S.10** Excitation dependent emission in 5 nm increments from 220 nm to 300 nm, in aqueous suspension.

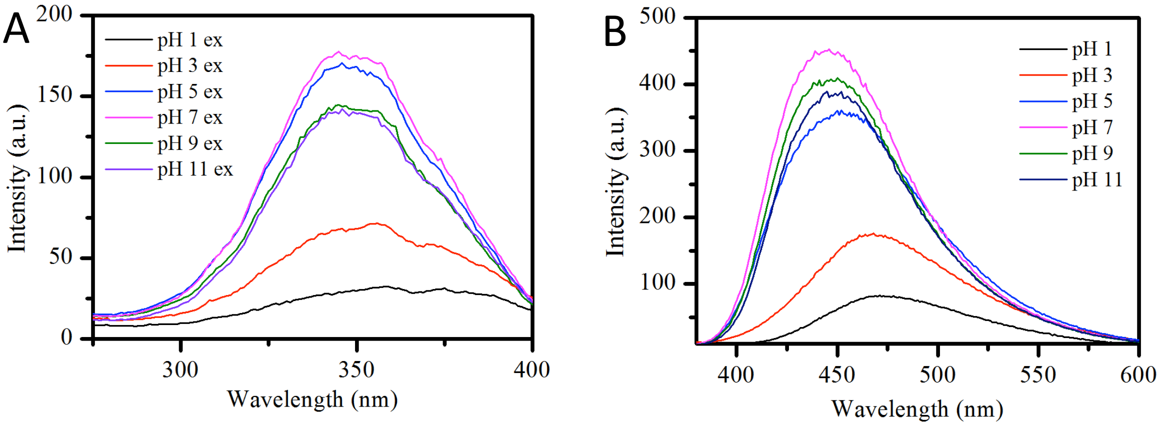


**Figure S.11** **A** Excitation spectra for each of the triplicate pH dependent emission studies in aqueous buffer solutions for emission at 450 nm **B** Emission spectra for each of the triplicate pH dependent emission studies in aqueous buffer solutions excited at 350 nm.

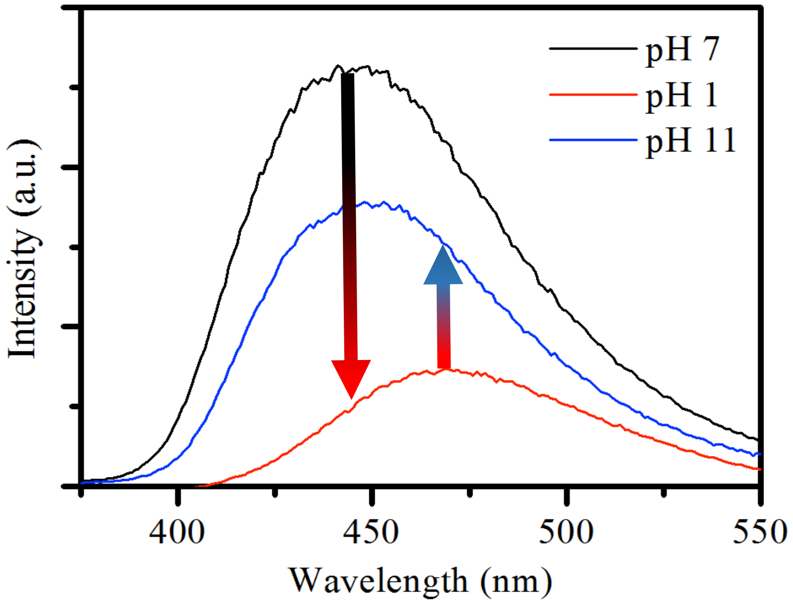


**Figure S.12** Emission spectra of carbon fibers in aqueous suspension with the pH being sequentially changed to show switchable pH dependent emission. From pH 7 to pH 1 to pH 12.

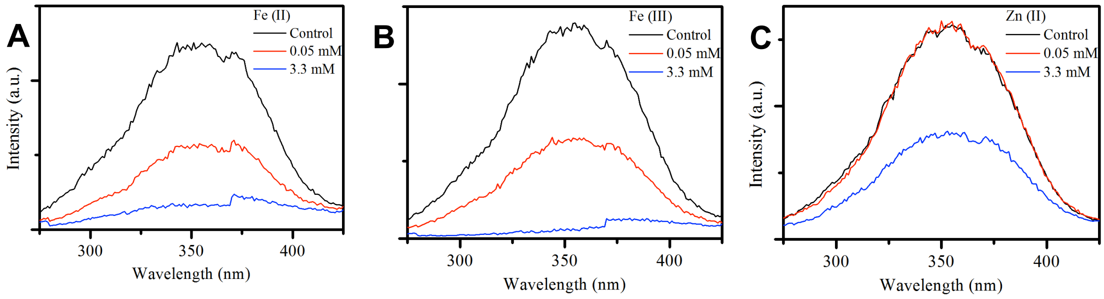


**Figure S.13** Excitation spectra of carbon fibers (for 450 nm emission) under various concentrations of metal ions (Fe(II) Fe(III) and Zn(II)) in water, showing no shift under various concentrations of quenching.

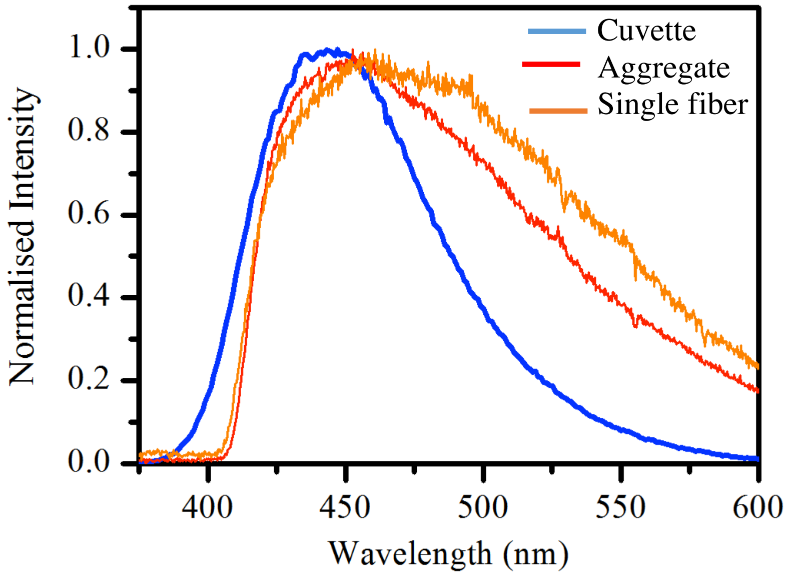


**Figure S.14** Overlay of emission spectra in suspension, deposited few fibers (aggregate) and deposited single fiber.

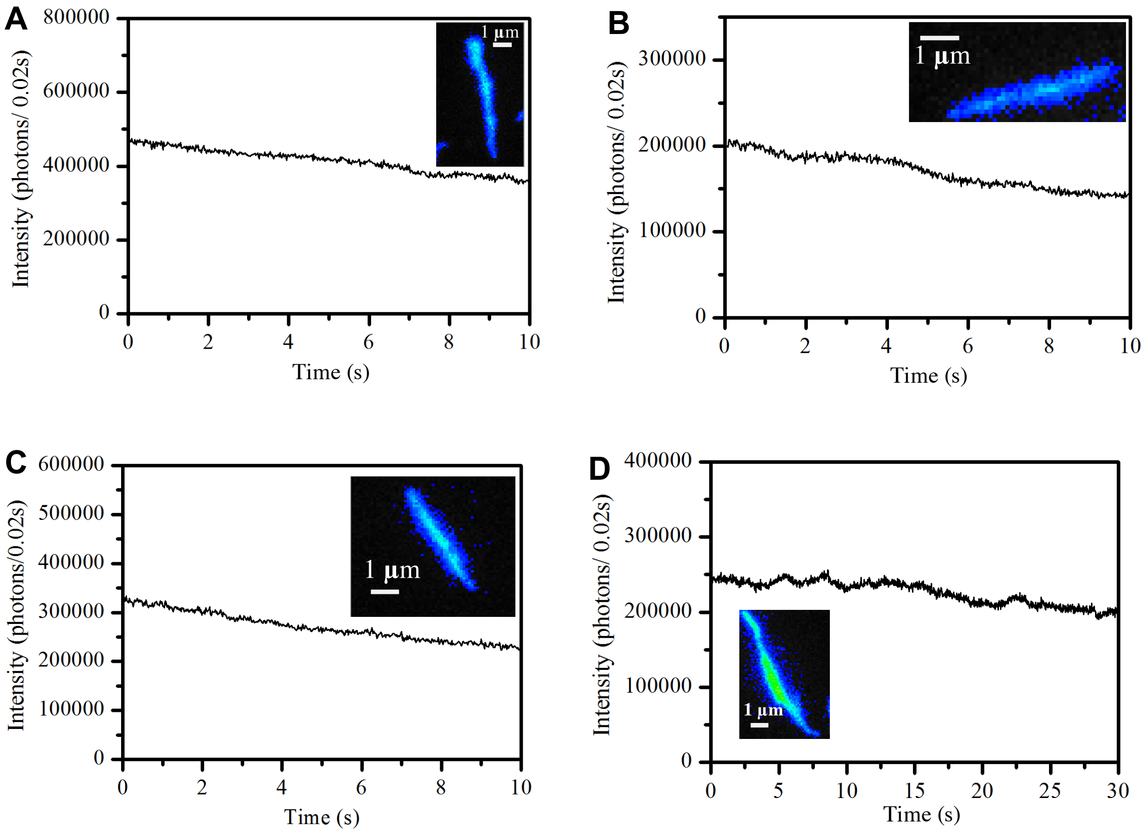


**Figure S.15** Photostability in water of a range of single fibers.

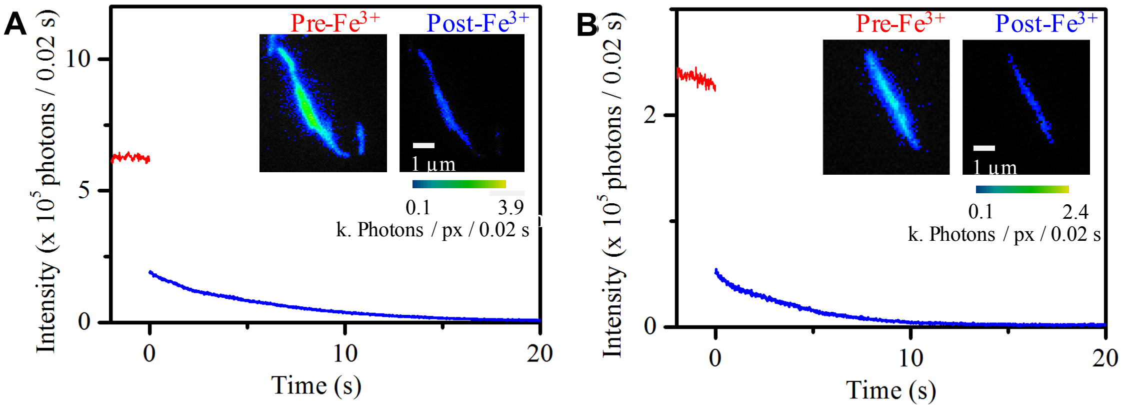


**Figure S.16** Examples of single fiber quenching of luminescence of a carbon fiber deposited on glass and covered in water (red) and after the introduction of Fe(III) (blue), the resulting concentration being 1 mM.

## Tables

| Table S1: Values used for percentage yield calculations | |
|---|---|
| **Number of pores per membrane** | $1.84x10^{9} \pm 0.22x10^{9}$ |
| **Pore length** | 60 μm (as listed) |
| **Pore diameter** | 0.24 ± 0.03 μm |
| **Pore volume** | $1.9x10^{-18} \pm 0.23x10^{-18}$ $m^3$ |
| **Volume per membrane** | $3.5x10^{-9} \pm 0.4x10^{-9}$ $m^3$ |
| **Density of amorphous carbon** | 2 $g.cm^{-3}$ |
| **Mass carbon per membrane** | 6.9 ± 0.8 $mg.mL^{-1}$ |
| **Actual mass of carbon** | 0.7 ± 0.4 $mg.mL^{-1}$ |

| Table S2: Carbon fiber dimensions | | | |
|---|---|---|---|
| | **SEM**<br>**nm** | **AFM**<br>**μm** | **Bright Field**<br>**μm** |
| **Carbon fiber diameter** | 237.7 ± 39.2<br>*n* = 110 | 206.9 ± 40.5<br>*n* = 54 | - |
| **Carbon fiber length** | - | - | 10.5 ± 5.3<br>*n* = 1250 |
| **Membrane pore opening diameter** | 212.9 ± 30.6<br>*n* = 1087 | - | - |

| **Table S3: Peak analysis for XPS** | | |
|---|---|---|
| **Spectrum** | **Peak position (eV)** | **Assignment** |
| **XPS Full spectrum** | 284.8<br>400.6<br>531.9 | C<br>N<br>O |
| **XPS C1S** | 284.9<br>286.3<br>288.1 | C-C/C=C<br>C-O/C-N<br>C=O |
| **XPS O1S** | 531.5<br>532.8 | C-O<br>C=O |
| **XPS N1S** | 400.6<br>401.3 | Amino N<br>Pyrrolic N |

| **Table S4: Peak analysis for IR** | | |
|---|---|---|
| **Peak position ($cm^{-1}$)** | **Assignment** | **Description** |
| 3356 | O-H | Alcohol stretch, H-bonded – broadened |
| 3293 | N-H | Aliphatic amine stretch (symmetric) |
| 2931 | C-H | Alkane stretch (asymmetric) |
| 2850 | O-H | Acid stretch |
| 1635 | C=O | Amide stretch |
| 1570 | C=C | Aromatic stretch |
| 1404 | C-H | Alkane bend |
| 1258 | C-O / C-N | Ether stretch /Amine stretch |
| 1111 | C-O | Alcohol stretch |

| **Table S5: Suspension lifetime measurements** | | | | |
|---|---|---|---|---|
| $\tau_1$ | % | $\tau_2$ | % | $\chi^2$ |
| 1.02 ns | 69 | 9.01 ns | 31 | 15.44 |

| Table S.6 Optical properties of various synthesis of similarly prepared carbon dots | | | | | | | |
|---|---|---|---|---|---|---|---|
| **Precursors** | **Method of carbonization** | **Diameter (nm)** | **Emission peak (nm)** | **Stokes shift (nm)** | **Quantum yield** | **Fluorescence lifetime** | **Ref.** |
| Citric acid / PEI | Microwave | 200 nm | 450 nm | 100 nm | 5% | 1.02 ns (69 %) 9.01 ns (31 %) | This work |
| Citric acid/ EDA | Microwave hydrothermal | 2.2-3.0 nm | 460 nm | 100 nm | 30.2% | 15.90 ns | 1 (57) |
| Citric acid/ PEI | Microwave hydrothermal | 12 nm | 445 nm | 95 nm | 30% | 11.55 0.22 ns 3.07 0.19 ns 0.54 0.06 ns | 2 (69) |
| Citric acid/ EDA/ $Mg(OH)_2$ | hydrothermal | 0.8-2.8 nm | 437 nm | 77nm | 83% | N/A | 3 (65) |
| Citric acid/ EDA | hydrothermal | 2-6 nm | 443 nm | 83 nm | 80% | 7.8 ns (ca. 20 %) and 0.9 ns (ca. 80 %) | 4 (58) |
| Citric acid/ DETA/ o-PA | pyrolysis | 2 nm | 450 nm | 90 nm | 64% | 35.3 ps (43%) 57.6 ps (57%) | 5 (70) |
| Citric acid/ EDA | hydrothermal | 3.5 nm | 450 nm | 110 nm | 22% | 0.19 ns 1.67 ns 6.52 ns | 6 (71) |
| Citric acid/ EDA (or TEA) | hydrothermal | N/A | 440 nm | 100 nm | 52% (or 7%) | 7.3 ns | 7(72) |
| Citric Acid/ Urea | hydrothermal | <1 nm | 440 nm | 80 nm | 35.1% (at pH 7) | N/A | 8 (67) |
| Citric Acid/ LEPI | hydrothermal | 1-3 nm | 450 nm | 130 nm | 37.4% | N/A | 9 (34) |

*Reference number for main text in parenthesis.

| | **Table S7. pH dependent emission and excitation shifts.** | |
|---|---|---|
| **pH** | **λ Of maximum excitation (nm)** | **λ of maximum emission(nm)** |
| 1 | 359 | 471 |
| 3 | 356 | 467 |
| 5 | 346 | 455 |
| 7 | 345 | 447 |
| 9 | 345 | 449 |
| 11 | 346 | 449 |

| **Table S8: Deposited lifetime measurements** | | | | |
|---|---|---|---|---|
| $\tau_1$ | % | $\tau_2$ | % | $\chi^2$ |
| 1.35 ns | 76.3 | 5.3 ns | 23.7 | 0.97 |

| Table S. 9 Literature comparison (for carbon dots) of metal ion quenching trends. | | | | | | |
|---|---|---|---|---|---|---|
| **Precursors** | **Method of carbonization** | **Sensitivity to** | **Percentage quenched** | **Conc.** | **Comments /further analysis** | **Ref.** |
| Citric acid / BPEI | Hydrothermal | Cu(II), Hg(II) | 75% 25% | LOD Cu(II) 6 nM | Linear range from 0-11μM shown (pH4) for Cu(II). Detection in spiked river water. | 10 (80) |
| Citric acid/ EDA | Hydrothermal | Fe(III) Fe(II),Cu(II), Zn(II), Co(II), Hg(II) | 99% Others mentioned as 'less" | LOD 1ppm (spectra shown) | Lifetime changes show dynamic quenching | 4 (58) |
| Citric acid/ EDA | Hydrothermal (bomb) | Hg(II) | 70% | 20 μM | Full quenching by 12 μM, linear range (decrease) shown. Turns back on after EDTA | 11 (81) |
| Citric acid/ urea | Hydrothermal (bomb) | Be(II), Fe(III) | 96% 98% | $10^{-3}$M | Linear range from 0-1mM and 0.1 mM shown (respectively). Fe (III) trend is similar to this work) | 12 (82) |
| Citric acid/ PEG-diamine | Bomb No solvent | Fe(III), Fe(II) | 80% 30% | 500 μM | In cell detection | 13 (83) |
| Citric Acid | Heating mantle. No solvent, NaOH addition | $Cl_2$ | 75% | LOD o.o5 μM | Real tap water samples | 14 (84) |
| Citric Acid | Thermal pyrolysis | Fe (III), Hg(II), Pb(II) | 80% 70% 50% | LOD: 2.8 μM 5.7 μM 7.2 μM, | Sensitivity dependant on buffer systems | 15 (85) |
| Rice | Microwave | Fe (III) Zn (II) | 90% -150% | LOD: 0.78×10−7 M 1.63×10−7 M | Increase of emission seen in Zn(II) | 16 (78) |

*Reference number for main text in parenthesis.

**Bibliography**


1 X. Zhai, P. Zhang, C. Liu, T. Bai, W. Li, L. Dai and W. Liu, *Chem. Commun.*, 2012, **48**, 7955.

2 A. Salinas-Castillo, M. Ariza-Avidad, C. Pritz, M. Camprubí-Robles, B. Fernández, M. J. Ruedas-Rama, A. Megia-Fernández, A. Lapresta-Fernández, F. Santoyo-Gonzalez, A. Schrott-Fischer and L. F. Capitan-Vallvey, *Chem. Commun.*, 2013, **49**, 1103.

3 F. Li, C. Liu, J. Yang, Z. Wang, W. Liu and F. Tian, *RSC Adv.*, 2014, **4**, 3201–3205.

4 S. Zhu, Q. Meng, L. Wang, J. Zhang, Y. Song, H. Jin, K. Zhang, H. Sun, H. Wang and B. Yang, *Angew. Chemie Int. Ed.*, 2013, **52**, 3953–3957.

5 M. K. Barman, B. Jana, S. Bhattacharyya and A. Patra, *J. Phys. Chem. C*, 2014, **118**, 20034–20041.

6 N. Dhenadhayalan, K.-C. Lin, R. Suresh and P. Ramamurthy, *J. Phys. Chem. C*, 2016, **120**, 1252–1261.

7 J. Schneider, C. J. Reckmeier, Y. Xiong, M. von Seckendorff, A. S. Susha, P. Kasák and A. L. Rogach, *J. Phys. Chem. C*, 2017, **121**, 2014–2022.

8 W. Wang, C. Damm, J. Walter, T. J. Nacken and W. Peukert, *Phys. Chem. Chem. Phys.*, 2016, **18**, 466–475.

9 J. Liu, X. Liu, H. Luo and Y. Gao, *RSC Adv.*, 2014, **4**, 7648.

10 Y. Dong, R. Wang, G. Li, C. Chen, Y. Chi and G. Chen, *Anal. Chem.*, 2012, **84**, 6220–6224.

11 F. Yan, Y. Zou, M. Wang, X. Mu, N. Yang and L. Chen, *Sensors Actuators B Chem.*, 2014, **192**, 488–495.

12 X. Li, S. Zhang, S. A. Kulinich, Y. Liu and H. Zeng, *Sci. Rep.*, 2015, **4**, 4976.

13 H. Zhang, Y. Chen, M. Liang, L. Xu, S. Qi, H. Chen and X. Chen, *Anal. Chem.*, 2014, **86**, 9846–9852.

14 Y. Dong, G. Li, N. Zhou, R. Wang, Y. Chi and G. Chen, *Anal. Chem.*, 2012, **84**, 8378–8382.

15 C. Li, W. Liu, Y. Ren, X. Sun, W. Pan and J. Wang, *Sensors Actuators B Chem.*, 2017, **240**, 941–948.

16 N. Dhenadhayalan and K.-C. Lin, *Sci. Rep.*, 2015, **5**, 10012.
